# Degradation of Methylparaben Using Optimal WO_3_ Nanostructures: Influence of the Annealing Conditions and Complexing Agent

**DOI:** 10.3390/nano12234286

**Published:** 2022-12-02

**Authors:** M. Cifre-Herrando, G. Roselló-Márquez, D. M. García-García, J. García-Antón

**Affiliations:** Ingeniería Electroquímica y Corrosión (IEC), Instituto Universitario de Seguridad Industrial, Radiofísica y Medioambiental (ISIRYM), Universitat Politècnica de València, C/Camino de Vera s/n, 46022 Valencia, Spain

**Keywords:** photoelectrocatalysis, WO_3_ nanostructures, degradation, methylparaben, endocrine disruptors

## Abstract

In this work, WO_3_ nanostructures were synthesized with different complexing agents (0.05 M H_2_O_2_ and 0.1 M citric acid) and annealing conditions (400 °C, 500 °C and 600 °C) to obtain optimal WO_3_ nanostructures to use them as a photoanode in the photoelectrochemical (PEC) degradation of an endocrine disruptor chemical. These nanostructures were studied morphologically by a field emission scanning electron microscope. X-ray photoelectron spectroscopy was performed to provide information of the electronic states of the nanostructures. The crystallinity of the samples was observed by a confocal Raman laser microscope and X-ray diffraction. Furthermore, photoelectrochemical measurements (photostability, photoelectrochemical impedance spectroscopy, Mott–Schottky and water-splitting test) were also performed using a solar simulator with AM 1.5 conditions at 100 mW·cm^−2^. Once the optimal nanostructure was obtained (citric acid 0.01 M at an annealing temperature of 600 °C), the PEC degradation of methylparaben (C_O_ 10 ppm) was carried out. It was followed by ultra-high-performance liquid chromatography and mass spectrometry, which allowed to obtain the concentration of the contaminant during degradation and the identification of degradation intermediates. The optimized nanostructure was proved to be an efficient photocatalyst since the degradation of methylparaben was performed in less than 4 h and the kinetic coefficient of degradation was 0.02 min^−1^.

## 1. Introduction

Parabens are a group of chemicals widely employed as preservatives in pharmaceuticals and personal care products [1]. Due to their estrogenic activity, parabens are known as endocrine disruptor chemicals (EDC) [2]. Excessive use of these chemicals in consumer products may lead to potential health risks [3,4]. Methylparaben (MP) is one of the most characteristic and used parabens [5]. Consequently, MP was selected as the contaminant to be studied for this research. MP presents a hydroxyl group linked to an aromatic ring in para position, making the chemical more stable and thus, more difficult to degrade [6].

Wastewater treatment plants cannot effectively remove MP, as it has been detected in the environment [7,8]. Hence, new techniques using hydroxyl radical generation, called advanced oxidation processes (AOPs), are being studied [9,10,11,12]. For some years now, photoelectroctalysis (PEC) has been indicated as a promising method for removing emerging contaminants, such as EDC, from water [13,14,15]. The PEC technique combines electrolytic and photocatalytic processes. It consists in the generation of electron–hole pairs ($e_{CB}^{-}$/$h_{VB}^{+}$) on the surface of a semiconductor by means of the irradiation of the semiconductor with an energy greater than its bandgap. Furthermore, an external low bias is set in order to delay the recombination of $e_{CB}^{-}$/$h_{VB}^{+}$, enhancing the efficiency of the process [14].

An appropriate semiconductor for use as a photoanode in the PEC technique must have a proper bandgap, efficient $e_{CB}^{-}$/$h_{VB}^{+}$ separation and transport, and good photoelectrocatalytic properties and stability [16]. 

Tungsten trioxide (WO_3_) is raising attention for being used as a photoanode for PEC due to its favorable features: (i) suitable value of bandgap that enables absorption of part of the visible rays of the solar spectrum (≈2.6 eV, which corresponds to a wavelength of ≈480 nm), (ii) stability in acidic electrolytes (iii) excellent electrical conductivity and (iv) non-photoelectric corrosion [17,18,19]. Furthermore, previous studies from this group have proved that WO_3_ nanostructures are effective for removing different pesticides from water by PEC [16,18,20,21]. 

Anodization is a widely used method to form WO_3_ nanostructures, which enables to vary the morphology and dimensions of the electrode by changing the anodization conditions [19,22,23,24,25,26]. The addition of complexing agents to the electrolyte could be used to dissolve the tungsten in order to obtain solutions which will act as precursors in the formation of WO_3_ nanostructures. By tailoring the nanostructures, it is possible to improve their PEC properties. 

In previous work, the arrangement of WO_3_ nanostructures by the anodization of tungsten together with two different complexing agents, oxygen peroxide (H_2_O_2_) and citric acid, has been studied [27,28]. Consequently, the first motivation of the present work is to compare the best concentration of both complexing agents to select the optimal nanostructure. 

Another important factor affecting the PEC properties of the nanostructures is the temperature of the annealing treatment performed after their synthesis. It has been demonstrated that heat treatment at high temperatures transforms the amorphous structure of the WO_3_ into a crystalline one, changing also the morphology of the surface of the nanostructures [29]. In order to be used in PEC processes a crystalline structure of the nanostructures is needed [30,31]. Consequently, an investigation of the temperature for the synthesis of WO_3_ nanostructures with citric acid is also performed in this study in order to obtain enhanced WO_3_ nanostructures and apply them as a photoanode for MP degradation. 

To our knowledge, no PEC process using WO_3_ as a photoanode has been studied yet in the literature for MP degradation. Furthermore, the WO_3_ nanostructures are synthesized with a simple and non-expensive method. Hence, the objective of this study is to synthesize enhanced WO_3_ nanostructures with a very high electroactive surface area by the anodization method and test the excellent performance of the new WO_3_ nanostructure for use as a photoanode for the degradation of MP by PEC. On one hand, two important parameters in the synthesis of WO_3_ nanostructures are studied: the complexing agent and the annealing temperature. On the other hand, a solution with MP is degraded by PEC using the optimal WO_3_ nanostructure as a photoanode. 

## 2. Materials and Methods

### 2.1. Materials

Tungsten (W) bars of 99.5% purity with a diameter of 8 mm were used as working electrodes. Methanosulfonic acid (CH_4_O_3_S), hydrogen peroxide (H_2_O_2_), citric acid (C_6_H_8_O_7_) and sulphuric acid (H_2_SO_4_) were purchased from Sigma Aldrich. Furthermore, methanol (CH_3_OH), acetic acid (CH_3_COOH) and methylparaben standard (C_8_H_8_O_3_) were acquired, of high-performance liquid chromatography (HPLC) grade, from Sigma Aldrich (Saint Louis, MO, USA). Ultrapure water (18.2 MΩ·cm, Type 1 Water) was used for the preparation of all the solutions. 

### 2.2. WO_3_ Nanostructures Fabrication

In this work, the procedure followed to obtain the WO_3_ nanostructures was carried out by W electrochemical anodization under hydrodynamic conditions. The method was optimized by the authors in previous works [16,27,28]. 

First, before anodization, the W rod was conditioned. The W bar was polished using silicon carbide (SiC) papers of different grades (220, 500 and 4000) in order to obtain a mirror-like surface. Thereafter, the surface was washed with distilled water, sonicated in ethanol for 2 min and dried with air. Lastly, the sample was Teflon-coated to expose only a circular area of 0.5 cm^2^ to the electrolyte. 

For anodization, the polished W was used as a working electrode and a platinum foil as the counter electrode. During anodization, 20 V were applied for 4 h. The electrolyte was composed of 1.5 M methanosulfonic acid plus the complexing agent at 50 °C. The tungsten bar was assembled on a rotating disk electrode, and it was rotated continuously at a speed of 375 rpm. Two different complexing agents were used in order to find the optimal WO_3_ nanostructure: 0.05 M H_2_O_2_ and 0.1 M citric acid. The concentration chosen for each electrolyte was proved to be optimal in previous works [27,28]. 

After anodization, WO_3_ nanostructures were annealed at different temperatures (400 °C, 500 °C and 600 °C) in a flowing air atmosphere for 4 h to examine the effect of the annealing temperature on the nanostructure. 

### 2.3. Nanostructures Characterization

The morphology of the samples was characterized by field emission scanning electron microscope (FE-SEM). The equipment used was a Zeiss Ultra 55 Scanning Electron Miscroscope at an acceleration potential of 3 kV. Atomic force microscopy (AFM) in tapping mode was used to analyze the surface topography of the samples. The equipment used was a Bruker Multimode 8 (Bruker, Billerica, MA, USA). Furthermore, the crystallinity of the samples was analyzed using a confocal Raman laser microscope (Witec alpha300 R confocal Raman microscope, Ulm, Germany) with a neon laser of 488 nm with a power of 420 μW. 

To further study, the best obtained samples were also characterized by X-ray diffraction (XRD) using a Bruker D8AVANCE (Bruker, Billerica, MA, USA) diffractometer with a Cu radiation operating at 40 kV from 5 °C to 80 °C and X-ray photoelectron spectroscopy (XPS, K-ALPHA Thermo Scientific, Waltham, MA, USA). All XPS spectra were captured by Al-K monochromatized radiation (1486.6 eV) at 3 mA × 12 kV. The whole energy band was measured with scan pass energies of 200 eV and the specific elements at 50 eV. The experimental backgrounds were approximated by a smart background function and surface elemental composition was calculated from background subtracted peak areas. XRD permitted to obtain more detail about the crystalline structure of the samples, and XPS provided information about the electronic states of the nucleus and about the chemical status of WO_3_ nanostructures. 

Photoelectrochemical measurements under illuminated conditions were carried out using an Autolab PGSTAT302N potentiostat (Metrohm, Herisau, Switzerland) and a solar simulator (AM 1.5 conditions at 100 mW·cm^−2^). A three-electrode cell configuration was used, consisting of an Ag/AgCl (3 M KCl) reference electrode, a platinum tip as a counter electrode, and the WO_3_ nanostructures as a working electrode. The electrolyte used for all the photoelectrochemical measurements was 0.1 M H_2_SO_4_, to ensure the photostability of the electrode [32].

First, stability tests at 1 V_Ag/AgCl_ for 1 h were carried out for all the samples to check their photostability to photocorrosion. After that, photoelectrochemical impedance spectroscopy (PEIS) and Mott–Schottky tests were performed. On one hand, PEIS measurements were carried out, applying 1 V_Ag/AgCl_ in the frequency range varied from 100 kHz to 10 mHz with a 10 mV of amplitude. On the other hand, Mott–Schottky measurements were performed from 1 to 0.2 V_Ag/AgCl_ at a scan rate of 50 mV·s^−1^ and a frequency of 5 kHz with an amplitude signal of 10 mV. For stability, PEIS and Mott–Schottky tests of the area of the WO_3_ nanostructure exposed to the electrolyte was 0.5 cm^2^. 

After that, water-splitting tests were performed with the best samples, and photocurrent densities against potential curves were obtained. A potential sweep was scanned from 0.2 V_Ag/AgCl_ to 1.0 V_Ag/AgC_ at a scan rate of 2 mV·s^−1^ by pulsed light irradiation, 60 s light off and 20 s light on. 

### 2.4. PEC Degradation 

PEC degradation tests were performed in a photoelectrochemical quartz reactor of 14 mL in volume composed of a three-electrode electrochemical cell with the same configuration as the cell used for photoelectrohemical measurements (area of the anode = 0.5 cm^2^). A potentiostat provided 1V Ag/AgCl bias potential and a 1000 W Xenon lamp was used as a light source with an intensity of 100 mW/cm^2^. The distance from the lamp to the photoanode was 30 cm. 

The target solution to degrade was 10 ppm MP with 0.1M H_2_SO_4_ to assure acidic conditions and stability of the nanostructure. The PEC degradation was performed at room temperature, and the electrolyte was magnetically stirred. Samples were taken every hour during 24 h. 

The degradation process was followed by means of ultra high-performance liquid chromatography and mass spectrometry (UHPLC-MS-Q-TOF). The equipment used was an Agilent 1290 Infinity UHPLC equipment fitted with a C-18 analytical column (Agilent ZORBAX Eclipse Plus C18, 50 mm × 2.1 mm, 1.8 μm particle size). The temperature of the column was 30 °C, the flow rate used was 0.8 mL/min, and the injection volume was 2 μL. Mobile phases consisted of (A) water + 0.01% Acetic Acid (*v*/*v*) and (B) methanol. The analysis started with 20% mobile phase B. Then, the solvent B increased linearly to 90% in 25 min, was kept at 90% for 1 min, and the initial condition was restored in 0.5 min. The re-equilibration time was 1.5 min. The total run time was less than thirty minutes. 

The UHPLC system was coupled to a time-of flight mass spectrometer (MS-Q-TOF) fitted with an electrospray interface operated in negative ionization mode. The MS spectra were registered from 70 to 1200 m/z. The parameters of the MS-Q-TOF were the following: capillary voltage 4000 V; nebulizer pressure 45 psi; drying gas flow rate 11 L/min; gas temperature 355 °C; skimmer voltage 65 V; octopole rf 250 V; fragmentor voltage 90 V. Finally, data acquisition was performed by Agilent MassHunter Qualitative Analysis 10.0 Software. 

## 3. Results

### 3.1. FE-SEM

**Figure 1:**
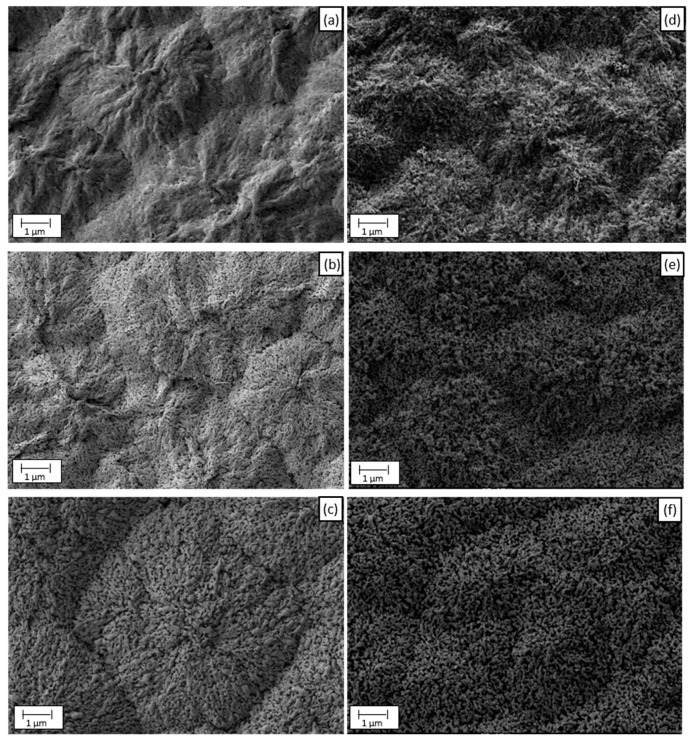
FE-SEM images of the WO_3_ nanostructures synthesized with different complexing agents and different temperatures: (**a**) 400 °C—0.05 M H_2_O_2_ (**b**) 500 °C—0.05 M H_2_O_2_ (**c**) 600 °C—0.05 M H_2_O_2_ (**d**) 400 °C—0.1 M citric acid (**e**) 500 °C—0.1 M citric acid and (**f**) 600 °C—0.1 M citric acid.

All samples were characterized morphologically by FE-SEM. Figure 1 shows different FE-SEM images of the WO_3_ nanostructures formed after the annealing treatment carried out at various temperatures (400 °C, 500 °C and 600 °C) in both complexing agents used during the synthesis. It can be observed that for all temperatures and complexing agents, small-sized nanoparticles were obtained. Furthermore, Figure 1 shows that for both complexing agents, as the temperature increases, the morphology of the nanostructures formed is much more defined. At 400 °C, the nanostructures form a spongy layer with nanostructures grouped in a mountain-shape and poorly defined, and as the temperature increases, the nanostructures are more homogenous and in a defined nanorod shape. Consequently, the annealing temperature had a significant effect on the morphology of the WO_3_ nanostructures. According to a previous work from the research group [33], the clear definition of the nanostructures at a higher temperature could be associated with a higher degree of their dehydration. Concerning the complexing agent used during the synthesis, the morphology obtained with both complexing agents was quite similar. However, in the case of H_2_O_2_, the change of morphology with temperature was more noticeable than in the citric acid nanostructures. Furthermore, comparing the best annealing temperature of both complexing agents—Figure 1c,f—it can be seen that “the mountains” formed by the nanostructures obtained with citric acid (f) were more compact than those obtained with H_2_O_2_ and the nanorods formed were smaller in this case. The similarity in morphology for both electrolytes is due to the fact that both act as bidentate complexing agents and the same formation mechanism of nanostructures is taking place [27,28,34,35,36,37].

### 3.2. Raman

**Figure 2:**
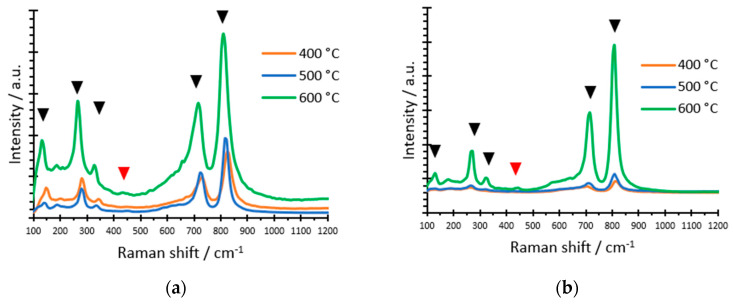
Raman spectra of the samples synthesized with (**a**) 0.05 M H_2_O_2_ and (**b**) 0.1 M citric acid and annealed at different temperatures (400 °C, 500 °C and 600 °C).

The Raman spectrum was used to assess the effect of the temperature of annealing and complexing agent on the composition and crystal structure of the samples. Figure 2a shows the Raman spectra for the samples synthesized with 0.05 M H_2_O_2_ and annealed at 400 °C, 500 °C and 600 °C, while Figure 2b shows the Raman spectra for the samples synthesized with 0.1M citric acid at the same temperatures. The shape of the spectra is similar for both complexing agents and the characteristic monoclinic WO_3_ peaks were appreciated in all cases: 125 cm^−1^, 273 cm^−1^, 327 cm^−1^, 715 cm^−1^and 822 cm^−1^ [34,38,39,40,41,42]. Although the characteristic peaks of crystalline WO_3_ were observed, some variations could be found based on the annealing temperature. The main characteristic was that, as the annealing temperature rose, the peaks had a higher intensity, indicating a higher crystallinity of the samples annealed at 600 °C. Furthermore, there are two bands (pointed by the red arrow in Figure 2) that are only seen in the 600 °C samples: 195 cm^−1^ and 434 cm^−1^. Those weak bands are also associated with crystalline tungsten oxides [34,41]. Consequently, it can be concluded that the annealing temperature is crucial, being that 600 °C is the optimal value for obtaining WO_3_ nanostructures with high levels of crystallinity and dehydration. 

### 3.3. Stability Test

**Figure 3:**
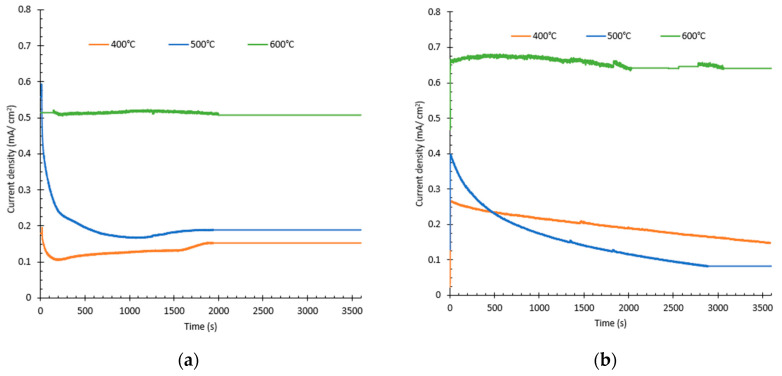
Stability test for (**a**) 0.05 M H_2_O_2_ and (**b**) 0.1M citric acid obtained under illumination at an applied potential of 1 V_Ag/AgCl_ in a 0.1M H_2_SO_4_ for 1 h.

For all the samples, stability tests were carried out to evaluate their photocurrent resistance under illumination. These results are shown in Figure 3. For 0.05 M H_2_O_2_, the sample annealed at 600 °C showed the highest photocurrent density (0.51 mA/cm^2^); moreover, it behaved in a stable way. For the samples annealed at 400 °C and 500 °C, at the beginning, the current decreased sharply, showing instability and then, they remained constant but showed a very low current (0.15 and 0.20 mA/cm^2^, respectively). For the samples synthesized with 0.1 M citric acid, the samples followed the same tendency as those synthesized with 0.05 M H_2_O_2_, although only the sample annealed at 600 °C was stable. The sample annealed at 600 °C had the highest photocurrent density (0.64 mA/cm^2^), while the samples synthesized at 400 °C and 500 °C apart from being unstable, showed lower values of photocurrent (0.20 and 0.10 mA/cm^2^, respectively). From these results, it can be concluded that the annealing temperature is a determining feature for obtaining nanostructures with optimal photo electrochemical properties, being that 600 °C is the best temperature. Furthermore, from the higher values of photocurrent density, it can also be concluded that in the WO_3_ nanostructures synthesized with citric acid in the electrolyte, the holes generated are transferred faster between the electrolyte and the nanostructures, and the e^−^/h^+^ pairs are kept separated for a longer time, enhancing the efficiency of the process [43,44]. Consequently, nanostructures synthesized with 0.1 M citric acid and annealed at 600 °C are optimal for use as photoelectrocatalysts. 

### 3.4. PEIS

**Figure 4:**
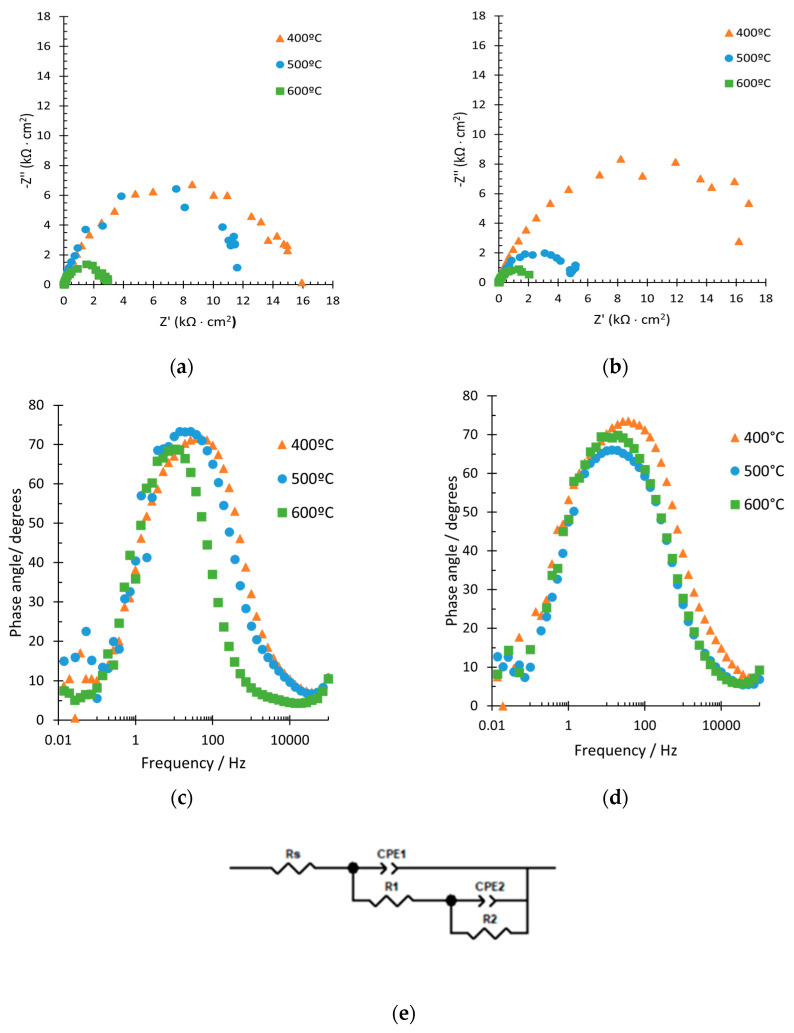
Nyquist plots of the samples annealed at different temperatures and synthesized with (**a**) 0.05 M H_2_O_2_ and (**b**) 0.1 M citric acid. Bode plots of the samples anodized with (**c**) 0.05 M H_2_O_2_ and (**d**) 0.1M citric acid and (**e**) equivalent circuit.

**Figure 5:**
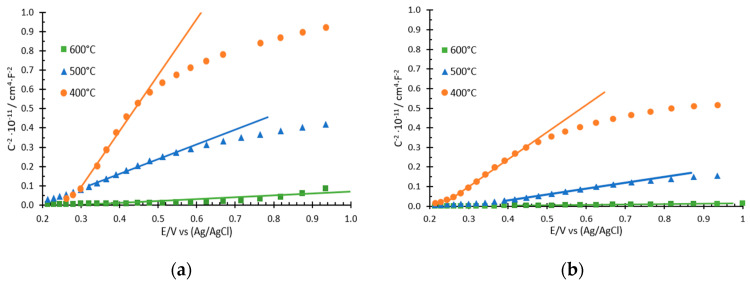
Mott–Schottky plots under illuminated conditions (simulated solar light AM 1.5) for the samples synthesized with (**a**) 0.05 M H_2_O_2_ and (**b**) 0.1 M citric acid at different annealing temperatures.

In addition to stability test, photoelectrochemical impedance spectroscopy (PEIS) tests under illuminated conditions were carried out in all the synthesized samples in order to further analyze their photoelectrochemical characteristics. Nyquist diagrams and Bode plots (Figure 4) were obtained from PEIS tests. Finally, Mott–Schottky (MS) analyses were also performed (Figure 5) to obtain the defects density of a sample. PEIS test allowed to study the influence of the temperature of annealing and the complexing agent on the mechanism of photocurrent generation and the photoelectrochemical events occurring in the WO_3_ nanostructures. 

The semicircle shown in the Nyquist diagram (Figure 4a,b) for the study of illuminated nanostructured semiconductors is associated with charge-transfer processes of the electron–hole pairs [45,46]. The hole transfer allows the medium to oxidize by forming species, such as hydroxyl radicals or oxidized organic matter. Thus, as the semicircle decreases, the photoelectrochemical response of the nanostructures improves [45,47]. From Figure 4a,b, it can be seen that in all cases, the circle decreases with increasing temperature. Although only a semicircle is visible at first sight in the Nyquist plots, at high frequencies there is a smaller semicircle. For the nanostructures synthesized with H_2_O_2_, the semicircle is similar for annealing temperatures of 400 °C and 500 °C, while it decreases drastically at 600 °C, indicating better photoelectrochemical properties. For the samples synthesized with citric acid, the semicircle was similar when the annealing was performed at 500 °C and 600 °C, and it was much higher at 400 °C. In this case, the photoelectrochemical response was better at 600 °C, which was confirmed with the morphological variations seen in the FESEM analysis (see Figure 1). Therefore, charge-transfer processes under illuminated conditions were favored at an annealing temperature of 600° for both complexing agents. 

From Bode phase diagrams (Figure 4c,d), for both complexing agents, H_2_O_2_ and citric acid, a wide peak was appreciated in all the annealing conditions, which is likely to be an indicator of the superposition of the two individual peaks [48]. The results are very similar in all cases, although for both complexing agents, it can be appreciated that the phase angle was lower for 600 °C, indicating more resistance, thus, better photoelectrochemical properties [45].

To quantitatively analyze the PEIS results, the electric circuit shown in Figure 4e is employed. The circuit is composed of one resistance (Rs) and two R-CPE time constants. The R_S_ is linked to the electrolyte resistance, the first time constant (R_1_-CPE_1_) to the resistance to the recombination of the electron–hole pairs and the second (R_2_-CPE_2_) to the resistance from WO_3_ to the electrolyte [45,46,47,49,50]. In this circuit, constant phase elements (CPE) are implemented as substitutes for capacitors to simulate the non-ideality of electrochemical capacitances.

The results of all the samples are shown in Table S1 (see Supplementary Material). Results from H_2_O_2_ electrolyte are obtained from [48]. From Table S1, it can be noticed that in all samples, R_1_ was always lower than R_2_, as it was predicted since R_1_ was not appreciated in Figure 4. Furthermore, R_2_ decreased with increasing temperature, regardless of the complexing agent used. Comparing the complexing agent, the value of R for the citric acid was always lower than for H_2_O_2_, indicating higher separation performance of photogenerated $e_{CB}^{-}$/$h_{VB}^{+}$ pairs and also a faster charge transfer of holes from the nanostructure to the electrolyte. 

Afterwards, MS analyses were also conducted in order to gather more information about the semiconducting properties of the WO_3_ nanostructures. Figure 5 shows that from all samples, MS plots have a linear region with a positive slope, which is a characteristic of n-type semiconductors [28]. Furthermore, this region is related to the presence of donor species, such as oxygen vacancies, in the crystalline structure of WO_3_ [48]. This slope is inversely proportional to the number of defects in the samples. Consequently, the lower the slope, the higher the defect number and therefore, the better the photoelectrocatalytic properties of the sample [31,51,52]. From Figure 5, it can be seen that, for both complexing agents, the slope is lower as the annealing temperature increases. 

Finally, through the positive slope of MS plots (Figure 5), the density of donors (N_D_) and the flat band potential (E_FB_) of the samples were obtained (Table S2). Following the tendency of the slopes, N_D_ increased as the annealing temperature increased. From 400 °C to 500 °C, the N_D_ slightly increased, and from 500 °C to 600 °C, it raised sharply. Moreover, in all cases, N_D_ was always higher for citric acid than for H_2_O_2_. A high number of defects resulted in higher mobility of the electron–hole pairs in the nanostructure, improving the photoelectrochemical performance of the anode. Regarding the E_FB_ values, they were all around 0.3 V (vs. Ag/AgCl) without seeing an influence of the complexing agent or the annealing temperature used. This value of around 0.30 V was expected, as it is characteristic for WO_3_ nanostructures according to the values found in the literature [53].

From all the results shown above, it can be concluded that while the heating temperature is a key factor for obtaining optimal nanostructures to use as photoanodes, both complexing agents used in this study had similar effects. Furthermore, it was demonstrated that the optimal annealing temperature is 600 °C. Consequently, for the following characterization of the samples, only samples annealed at 600 °C were studied.

### 3.5. XRD 

XRD characterization of the samples annealed at 600 °C was carried out to study the influence of the electrolyte on the crystallinity of the WO_3_ nanostructures. Figure S1 (see Supplementary Material) shows the XRD pattern of WO_3_ nanostructures, where all peaks (002), (020), (200), (112), (022), (220), and (221) can be associated to the monoclinic WO_3_ phase. For both electrolytes, the sharper peaks were (002), (020) and (220), indicating that both synthesized nanostructures belong to the monoclinic phase of WO_3_ [21,54,55,56,57,58].

### 3.6. AFM

The topography and the average roughness values (Ra) of the samples were obtained by atomic force microscopy (AFM). From Figure S2, it can be observed that both nanostructures presented a similar topography, manifesting a porous organization with clusters of grains of the order of nanometers. Furthermore, it can be seen that the sample synthesized with citric acid has a higher value of roughness than that one synthesized with H_2_O_2_, which could be beneficial for the adsorption of organic compounds [16]. Therefore, with AFM results, it can be affirmed that the roughness of the sample synthesized with citric acid is higher than that one synthesized with H_2_O_2_. Consequently, the porosity of the samples obtained with citric acid is also higher, indicating a larger surface area.

### 3.7. XPS

X-ray photoelectron spectroscopy (XPS) analysis was performed on the two samples annealed at 600 °C to provide insight into the chemical state of the samples. The XPS technique analyzes the electronic states of the nucleus and of the valence band of the WO_3_ samples in order to realize a qualitative and quantitative comparison of all the elements present in the nanostructures. The only elements found in both samples were tungsten and oxygen. 

**Figure 6:**
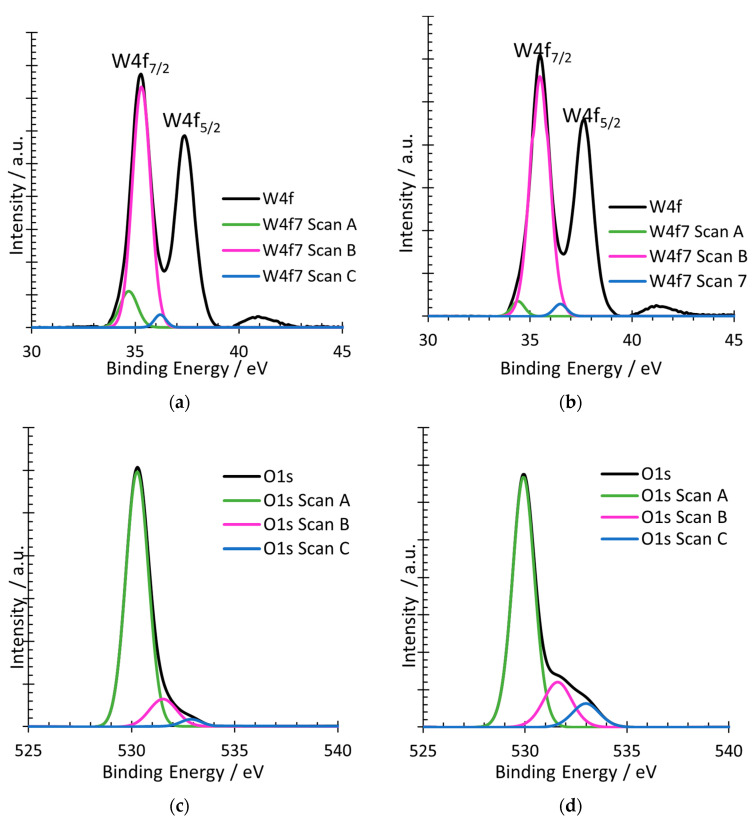
High resolution XPS spectra of peak W4f and O1s of the samples annealed at 600 °C and synthesized with different electrolytes: (**a**) W4f—H_2_O_2_ 0.05 M and (**b**) W4f—citric acid 0.1 M, (**c**) O1s—H_2_O_2_ 0.05 M and (**d**) O1s—citric acid 0.1 M.

The majority element detected in both samples in the XPS spectrum was tungsten. Figure 6a,b shows the XPS spectra of the peak W4f, which is associated with tungsten, for the samples anodized with (a) H_2_O_2_ and (b) citric acid. Figure 6a,b reveals the two characteristic peaks of tungsten, W4f_7/2_, and W4f_5/2_, with binding energies of 35.4 eV and 37.5 eV, respectively. Furthermore, the deconvolution of the first peak (W4f_7/2_) was carried out in order to associate the convoluted peaks with different states of tungsten. For both samples, three peaks linked with tungsten were detected. The first one, Scan A, appeared at 34.5 eV. This peak was linked to vacancies or defects in the samples and, particularly, with the photoelectrons generated by the W atoms close to the oxygen vacancies that have oxidation states less than +6 (WO_3-x_) [21,59]. The second one, Scan B, appeared at 35.4 eV. This peak corresponds to the generation of photoelectrons emitted by W atoms with oxidation state +6, i.e., when stoichiometric tungsten oxide is present [60]. The third one, Scan C, appeared at 36.4 eV. This peak, as Scan A, is also associated with defects in the nanostructures; specifically, this peak is attached to local variations in the energy of the vacuum level caused by superficial defects [59]. Figure 6a was obtained from the studies of [21].

Comparing both complexing agents, although they are very similar, some differences in Scan can be appreciated. Scan A for H_2_O_2_ is slightly higher than that for citric acid, meaning that nanostructures synthesized with H_2_O_2_ had more surface defects than those with citric acid. However, in this case, more surface defects do not correspond with better electrochemical properties since all results in the electrochemical tests were always better for citric acid than for H_2_O_2_. This is confirmed with the oxygen peak. 

The other element found in the samples by the XPS analysis was oxygen. Figure 6c,d shows the XPS spectra of the peak O1s, which is associated with oxygen, for the samples synthesized with (a) H_2_O_2_ and (b) citric acid. Figure 6c,d reveal the characteristics peak of oxygen, O1s, with a binding energy of 529.8 eV and its deconvolution into three different peaks. The first peak (Scan A) has a binding energy of 530 eV and corresponds to the oxygen atoms O_2_^−^ in the lattice. The second peak (Scan B) has a binding energy of 531.5 eV and is associated with the defects of the nanostructure. In particular, it is associated with hydroxyl radicals (^−^OH) that exist on the surface. The last peak (C), which appears at 532.9 eV, is also associated with the defects, but in this case, particularly to the species adsorbed and oxygen vacancies [61,62,63]. The peaks are very similar for both complexing agents, although Scan B and C are higher for citric acid. As these peaks are associated with the defects of the nanostructure, the higher the peaks, the more numerous the surface defects and the more numerous the oxygen vacancies. The results of the oxygen peaks are more consistent with the electrochemical tests than the tungsten peaks, being that the sample with citric acid is the one with more surface defects and, consequently, better photo electrochemical properties. 

### 3.8. Water Splitting Tests

**Figure 7:**
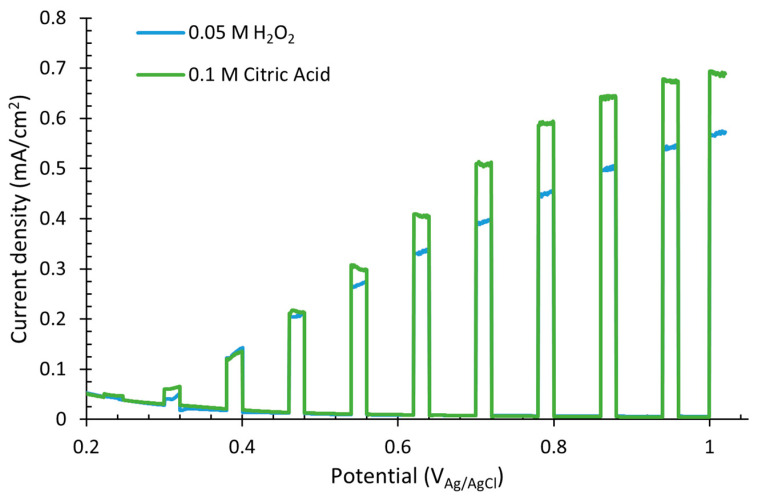
Water splitting curves of the WO_3_ nanostructures synthesized with different complexing agents measured in 0.1 M H_2_SO_4_ under AM 1.5 illumination.

The influence of the complexing agent used in the nanostructure synthesis on the photoelectrochemical (PEC) behavior of the nanostructures was also tested. Figure 7 shows the photocurrent density vs. potential curves, from where it can be seen that the sample anodized with citric acid presented higher photoresponse than that anodized with H_2_O_2_. This improvement (21% higher) of the PEC performance of WO_3_ nanorods synthesized with citric acid could be associated with the morphology of the nanostructures. As observed in the FESEM images (Figure 1c,f), the morphology of both samples annealed at 600 °C was well-defined, and the nanorods covered the whole surface of the sample, obtaining in both cases good response to the incident light and high photocurrent response. However, as the “mountains” obtained with the acid citric anodization (Figure 1f) were constituted by smaller nanorods and were more homogeneous than those with H_2_O_2_ (Figure 1c), the superficial area exposed was higher, consequently increasing the photocurrent density under illumination. This improvement can also be attributed to the better transport of electrons toward the metallic contact derived from the compact morphology of the nanostructure obtained with citric acid [28]. These results are also consistent with the results obtained with the Mott–Schottky test (Figure 3) and the N_D_ obtained in Table S2, as the N_D_ obtained for citric acid was higher than for H_2_O_2_ samples, indicating a higher number of defects, higher mobility of electron–hole pairs and consequently, better photocurrent response of the nanostructure with citric acid. 

It can be noticed from Figure 7 that for both cases, as the applied potential increased, the current density increased linearly. This indicates that the potential drop within the space charge layer is directly dependent on the bias potential, and consequently, the thickness of the layer and the strength of the electric field too [27,64].

### 3.9. Photoelectrocatalytic Degradation of Methylparaben

From the photoelectrocatalytic analysis of the samples, it can be concluded that the optimal sample to be used as a photoanode is the WO_3_ nanostructure synthesized with 0.1 M citric acid and annealed at 600 °C. Once the optimal nanostructure was determined, the photoelectrochemical degradation of the endocrine disruptor methylparaben was carried out. The degradation was performed over 24 h, being that 10 ppm was the initial concentration of the contaminant and samples were taken every hour. The samples were analyzed by UHPLC-Q-TOF/MS. 

Before the degradation, six standards of methylparaben from 0.5 to 10 ppm were analyzed to obtain a calibration curve, which will be used to calculate the concentration of the samples at different degradation times. Figure S3 shows the extracted ion chromatogram (EIC) of the standards for the m/z value 151.040, which corresponds to methylparaben [65]. Furthermore, Figure S3 also reveals the area of each standard and the retention time of the studied contaminant (5.5 min). Thus, with the area and the concentration of each standard, the calibration line was obtained, which is shown in the inset of Figure S3. The equation of the calibration line is the following (Equation (1)), which has an R^2^ value of 0.9944:

(1) $$Area\;\left(counts\right)=5800018.09\times concentration\;\left(\mathrm{ppm}\right)$$

**Figure 8:**
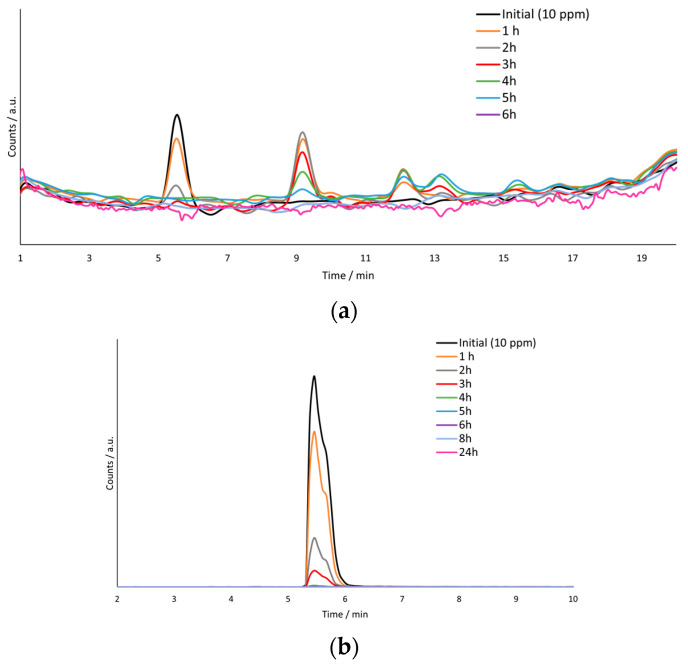
(**a**) TIC of the MP samples at different times of degradation. (**b**) EIC at a m/z of 154.0400 of the samples taken during the degradation.

After obtaining the calibration line, a PEC degradation of MP (C_o_ = 10 ppm) was performed for 24 h. Figure 8a shows the UHPLC-Q-TOF/MS TIC chromatograms of the samples taken every hour. In Figure 8a, it can be observed that three different peaks are clearly differentiated. The first peak, obtained at a retention time of 5.5 min, corresponds to MP. It can be observed that this peak decreases with time and practically disappears at 4 h, meaning that it is 100% degraded. This is confirmed by Figure 8b, which shows the EIC of the degradation samples for the m/z value associated to MP. On the other hand, both the second and third peaks, obtained at 9.2 and 12 min, correspond to intermediate degradation products of MP. 

First, the peak corresponding to methylparaben was analyzed. By obtaining the areas of each concentration and using the calibration line, the concentration of methylparaben as a function of time could be obtained (Table 1). From Table 1, it can be seen that, in only 4 h of degradation, the methylparaben was already 100% eliminated.

Once the concentration of each sample was calculated, the order of the degradation kinetics of the pollutant was determined, which is related to the velocity at which the contaminant is degraded. In this case, it can be approximated to pseudo-first order degradation kinetics, as there are two reactants present in the degradation process (MP and OH^−^ radicals) and the hydroxyl radicals remain approximately constant throughout the process. In Figure S4 (see Supplementary Material), it is represented the logarithm of the quotient between the concentration at each time and initial concentration versus time. The graph shown in Figure S4 presents a decreasing trend, indicating the effective PEC degradation of the contaminant; consequently, it can be affirmed that MP degradation follows a pseudo-first order reaction kinetics. The equation of the kinetics obtained is shown in Equation (2), where time is expressed in minutes and therefore, the kinetic coefficient has units of min^−1^:

(2) $$\ln\frac{C}{C_{o}}=-0.023\times t+0.631$$

Consequently, the kinetic coefficient of degradation of MP with WO_3_ nanostructures is 0.023 min^−1^. As in recent years several researchers have focused on the removal of endocrine disruptor chemicals; specifically for MP, in Table S4, there is a comparison of our results with those reported to date in the literature. Comparing our parameters with those obtained with other AOPs found in the literature, it can be affirmed that the WO_3_ nanostructures obtained in this study are very efficient for MP removal.

As mentioned previously, from the TIC chromatogram in Figure 8a, different peaks associated with methylparaben degradation intermediates were observed. Therefore, thanks to the chromatogram and the literature, several degradation intermediates could be identified. 

**Figure 9:**
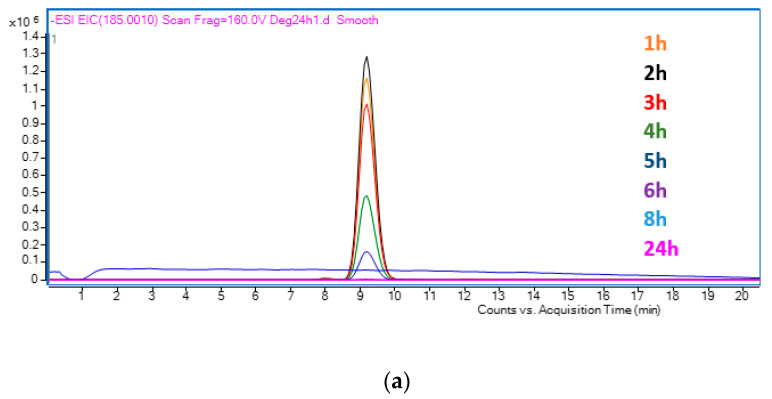

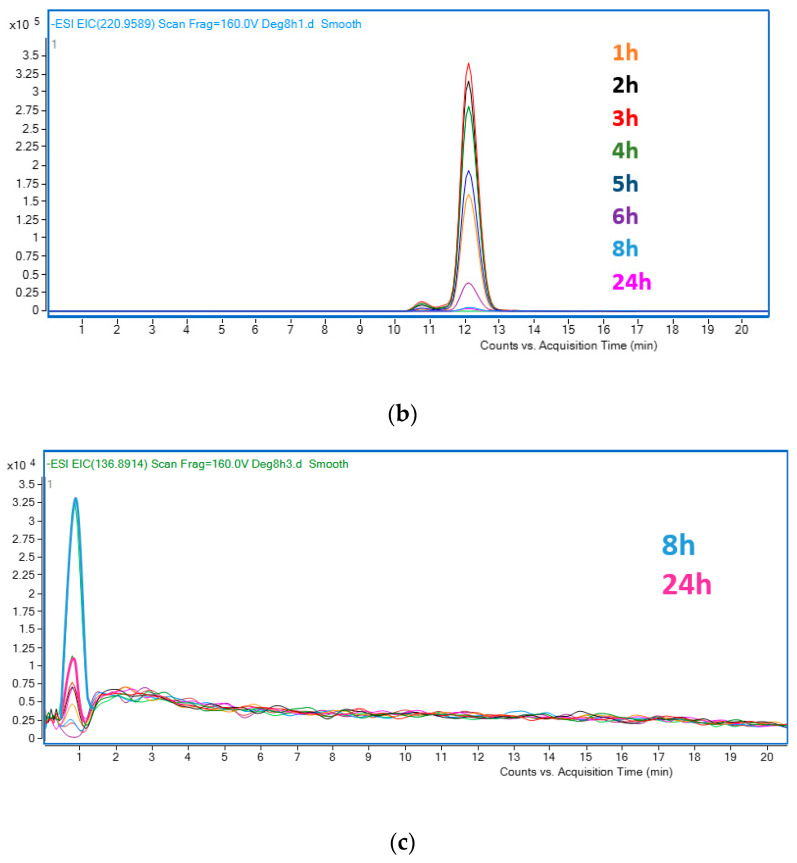
EIC of the different intermediates found in the PEC degradation of methylparaben: (**a**) m/z 185.0013 (**b**) m/z 220.9589 (**c**) 136.8914.

The m/z values found in the TIC chromatogram, apart from the m/z value associated to MP, were the following: 185.0013, 220.9616 and 136.8914. On the one hand, the first two values were obtained directly from the peaks associated with the TIC chromatogram in Figure 5. The value 185.0013 corresponds to the peak appearing at a retention time of 9.2 min and the value 220.96 corresponds to the peak at 12 min. On the other hand, the value of 136.894 was obtained from the literature [66]. Figure 9 shows the exact ion chromatogram (EIC) of the different intermediates of degradation found. Figure 9a shows the EIC of the m/z 185.0013; this value is associated with the compound tetra hydroxy benzoic acid [5]. Figure 9b shows the EIC of the third peak observed in the TIC, with a retention time of 12 min, and m/z value of 220.9589, which belongs to the compound diethyl phthalate [5]. Finally, Figure 9c shows the EIC of 136.8914, which was obtained from the literature [66], and it is associated with the chemical compound 4-hydroxybenzoic acid.

After carrying out this study, it could be stated that three reaction intermediates appeared: [A] tetra hydroxybenzoic acid, [B] diethyl phthalate and [C] 4-hydroxybenzoic acid.

Figure S5 shows the trend in concentration of each of these intermediates. Intermediates [A] and [B] appear when the degradation of MP starts, as they could be detected in the 1 h sample. Intermediate [A] reached its maximum concentration in the 2 h sample and then decreased until it was 100% eliminated at 6 h of degradation. Intermediate [B] followed the same tendency as intermediate [A]; it reached its maximum concentration in the 3 h sample and then decreased until it was 100% eliminated at 8 h of degradation. In contrast, compound [C] appeared in the 4 h degradation sample for the first time. Consequently, it was formed during the 3 and 4 h of degradation. Furthermore, it increased its concentration until it achieved its maximum at 24 h of degradation. Considering that intermediate [C] appears only after 3 h of degradation, it could be a product of one of the other two intermediates ([A] or [B]). Consequently, the two routes of degradation converged in compound [C], which is the final product of degradation. 

**Figure 10:**
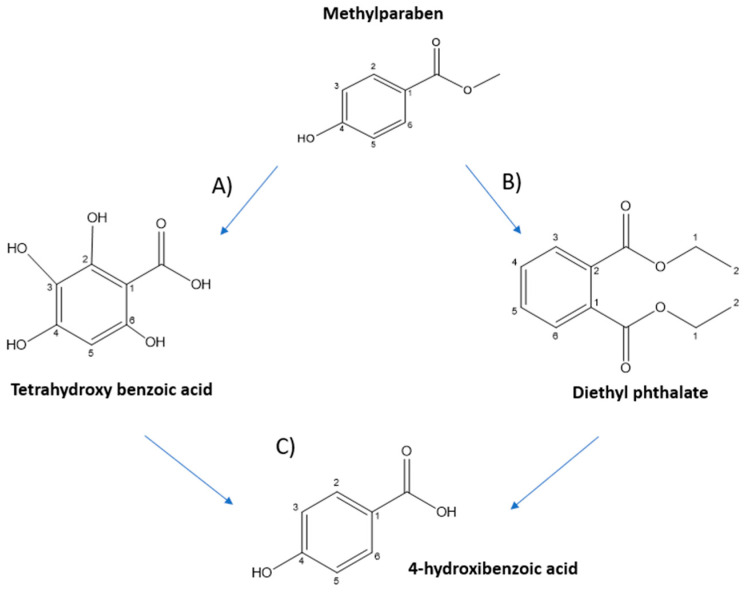
Degradation route of methylparaben and intermediates found. (**A**) Tetrahydroxy benzoic acid, (**B**) diethyl phthalate and (**C**) 4-hydorxybenzoic acid.

After studying the concentration of each intermediate of degradation, the degradation pathway for MP is proposed in Figure 10. In this pathway two degradation routes are proposed: (A) attack of the hydroxyl radical on methylparaben by hydroxylation of the aromatic ring and hydrolysis of the ester to carboxylic acid, and (B) formation of diethyl phthalate by various reaction mechanisms, such as dehydroxylation and the addition of alkyl groups to methylparaben. Furthermore, both intermediates can be transformed into 4-hydroxybenzoic. From [A] to [C], dehydroxylation takes place, and from [B] to [C], hydroxylation of the ring, double hydrolysis of the ester and a successive decarboxylation of the ester.

Table 2 shows the characteristics of the different intermediates found in the PEC degradation of methylparaben. 

Finally, as after 24 h of degradation, the only compound detected was 4-hydroxybenzoic acid, different standards of 4-hydroxybenzoic acid were analyzed at the UHPLC/MS in order to obtain a calibration line and to be able to quantify the concentration of this compound during the degradation. Figure S6 shows the calibration line obtained with the standards, and from the EIC of the compound shown in Figure 9c, the concentration of 4-hydroxybenzoic acid at each time of degradation could be obtained, shown in Table S3. From this data, it could be affirmed that methylparaben was degraded in 4 h, and the final product of the degradation was 4-hydroxybenzoic acid at a concentration of 2.60 ppm. 

From all these results, it can be concluded that the WO_3_ photoanode, obtained using citric acid as a complexing agent and annealed at 600 °C is an excellent photoelectrocatalyst, as it is possible to degrade methylparaben in less than 4 h. 

## 4. Conclusions

The aim of this work was to study the effect of the complexing agent and the annealing temperature in the synthesis of WO_3_ nanostructure in order to use them as a photoanode for the PEC degradation of an endocrine disruptor chemical, methylparaben. 

All results showed that the annealing temperature is a crucial factor for obtaining enhanced nanostructures of WO_3_ (high crystallinity, high superficial area and less resistance to charge transfer), with 600 °C being the optimal temperature. While the heating temperature is a key factor for obtaining WO_3_ nanostructures with favorable photoelectrochemical properties, the complexing agent is not that determinant. However, a further study of the complexing agent was carried out, and from the water-splitting test, it was clearly seen that nanostructures synthesized with citric acid had better photoelectrochemical properties than those with H_2_O_2_. 

On the other hand, the PEC degradation of MP was carried out with the optimal WO_3_ nanostructure, synthesized with 0.1 M citric acid and annealed at 600 °C, and it was shown that the contaminant could be 100% degraded in less than 4 h, which demonstrates the outstanding properties of WO_3_ nanostructures as a photoanode for the PEC degradation of MP. Furthermore, with the use of UHPLC-MS-Q-TOF a degradation pathway for the contaminant could also be proposed, and three intermediates of degradation were found. 

With these results, it can be affirmed that WO_3_ nanostructures synthesized with 0.1 M citric acid and annealed at 600 °C present an excellent behavior to degrade this type of endocrine disruptor. 

## Figures and Tables

**Table 1:** Concentration (ppm) and degradation (%) of MP during the degradation.

Time (h)	Area (Counts)	Concentration (ppm)	Degradation (%)
0	55,747,948	10.00	0.00%
1	36,171,413	6.24	37.64%
2	10,440,169	1.80	81.59%
3	3,693,070	0.64	93.44%
4	328,399	0.06	99.42%
5	88,928	0.02	99.84%
6	65,237	0.01	99.88%
8	0	0.00	100.00%
24	0	0.00	100.00%

**Table 2:** Characteristics of the intermediates of degradation of methylparaben.

Compound Identification	Compound	Molecular Formula	Retention Time (min)	m/z Value
A	Tetrahydroxy benzoic acid	C_7_H_6_O_6_	9.2	185.0013
B	Diethyl phthalate	C_12_H_14_O_4_	11	220.9586
C	4-Hydroxibenzoic acid	C_7_H_6_O_3_	0.9	136.8914

## Data Availability

Not applicable.

## Supplementary Materials

The following supporting information can be downloaded at: https://www.mdpi.com/article/10.3390/nano12234286/s1, Table S1: Resistance values of the WO_3_ samples with different electrolyte and annealing temperatures; Table S2: Density of donors (N_D_) and flat band potential (E_FB_) of the different samples; Figure S1: XRD plots of the samples anodized with 0.05 M H_2_O_2_ and 0.1M citric acid at 600 °C; Figure S2. Three-dimensional AFM images and roughness value (Ra) of the nanostructures annealed at 600 °C and synthesized with different electrolytes: (a) H_2_O_2_ 0.05 M and (b) citric acid 0.1 M; Figure S3: EIC at the m/z value 151.0400 of the standards of MP and inset of the calibration line; Figure S4: Degradation kinetics of MP; Figure S5: Concentration of all the intermediates in different times during the MP degradation: (a) intermediate A, (b) intermediate B, (c) intermediate C. Figure S6: Standards of the 4-hydroxybenzoic acid (m/z = 136.8914). Table S4: Concentration (ppm) of 4-hydroxybenzoic acid during the degradation of MP. Ref [67,68,69,70,71,72,73] are cited in the supplementary materials.
