# Supplementary material for: Degradation of Methylparaben Using Optimal WO3 Nanostructures: Influence of the Annealing Conditions and Complexing Agent
Source: Nanomaterials (Basel). 2022 Dec 2;12(23):4286. doi: 10.3390/nano12234286 (PMC9740506; doi:10.3390/nano12234286)
Supplement: Supplementary file 1 [file nanomaterials-12-04286-s001.zip › nanomaterials-2060266-supplementary.pdf]

SUPPLEMENTARY MATERIAL

Degradation of Methylparaben Using Optimal WO_3 Nanostructures: Influence of the Annealing Conditions and Complexing Agent

M. Cifre-Herrando, G. Roselló-Márquez, D. M. García-García and J. García-Antón *

Ingeniería Electroquímica y Corrosión (IEC), Instituto Universitario de Seguridad Industrial, Radiofísica y Medioambiental (ISIRYM), Universitat Politècnica de València, C/Camino de Vera s/n, 46022, Valencia, Spain
* Correspondence: jgarciaa@iqn.upv.es

Table S1. Resistance values of the WO_3 samples with different electrolyte and annealing temperatures.

Complexing Agent	Annealing Temperature / °C	R_S / Ω·cm²	R_1 / Ω·cm²	R_2/ Ω·cm²
H_2O_2 [49]	400	29 ± 3	159 ± 11	11.8 ± 1.6
	500	44 ± 9	107 ± 26	7.6 ± 1.3
	600	28 ± 6	26 ± 7	3.0 ± 0.9
Citric Acid	400	20±3	43±9	20.1±2.0
	500	17±2	5±1	5.3±1.4
	600	6±1	1±0.9	2.1±0.8

Table S2. Density of donors (N_D) and flat band potential (E_{FB}) of the different samples.

Complexing Agent	Annealing Temperature / °C	$N_D \cdot 10^{19}$/ cm³	E_{FB} / V
H_2O_2 [49]	400	0.4	0.320
	500	1.5	0.326
	600	28.5	0.312
Citric Acid	400	2.0	0.227
	500	9.4	0.305
	600	33.3	0.309

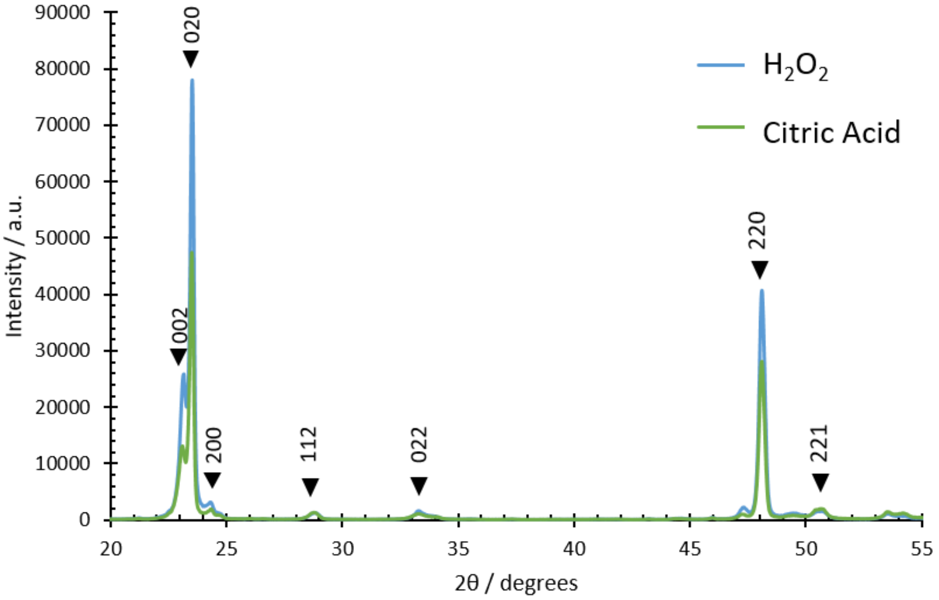

Figure S1. XRD plots of the samples anodized with 0.05 M H_2O_2 and 0.1M Citric acid at 600 °C.

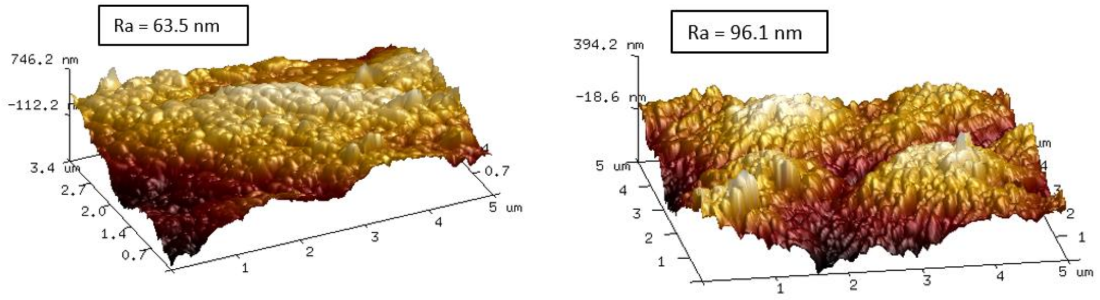

(**a**) (**b**)

Figure S2. Three-dimension AFM images and roughness value (Ra) of the nanostructures annealed at 600 °C and synthesised with different electrolytes: (**a**) H_2O_2 0.05 M and (**b**) Citric Acid 0.1 M.

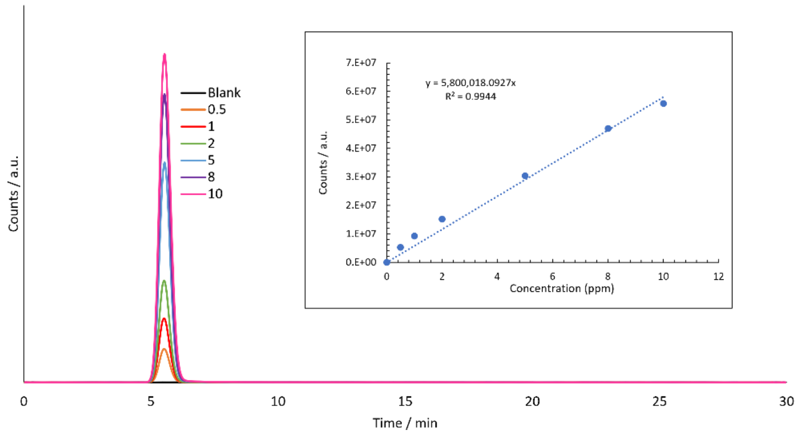

Figure S3. EIC at the m/z value 151.0400 of the standards of MP and inset of the calibration line.

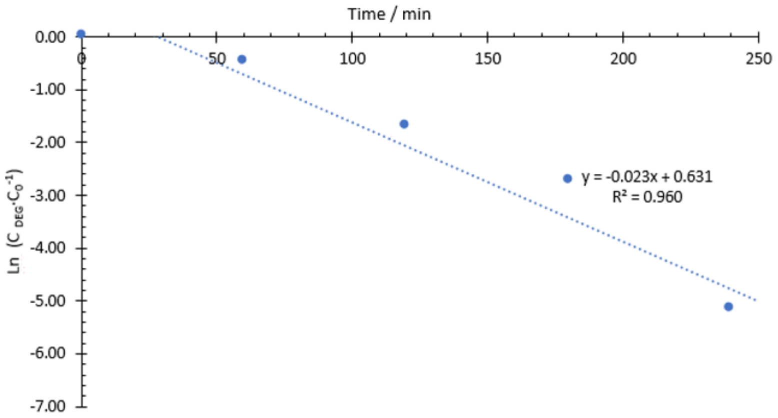

Figure S4 . Degradation kinetics of MP.

Table S3. Comparison of the kinetic coefficient with literature.

Technique of degradation	Initial concentration of MP (ppm)	K (min-1)	References
SPEF (BDD)	45	0.019	[67]
EO (BDD)	100	0.0029	[68]
SPEF	1	75% - 360 min	[69]
SECh (BDD)	100	0.0069	[70]
PC (TiO2)	10	100% -240 min	[71]
SECh (BDD)	100	0.024	[72]
PC (TiO2)	100	0.018	[73]

Abbreviations: BDD: Boron-doped diamond; EF: Electro-Fenton; EO: Electro-oxidation; PC: Photocatalysis; PEF: Photoelectro-Fenton, SECh: Sonoelectrochemical; SPEF: Solar photooelectro-Fenton.

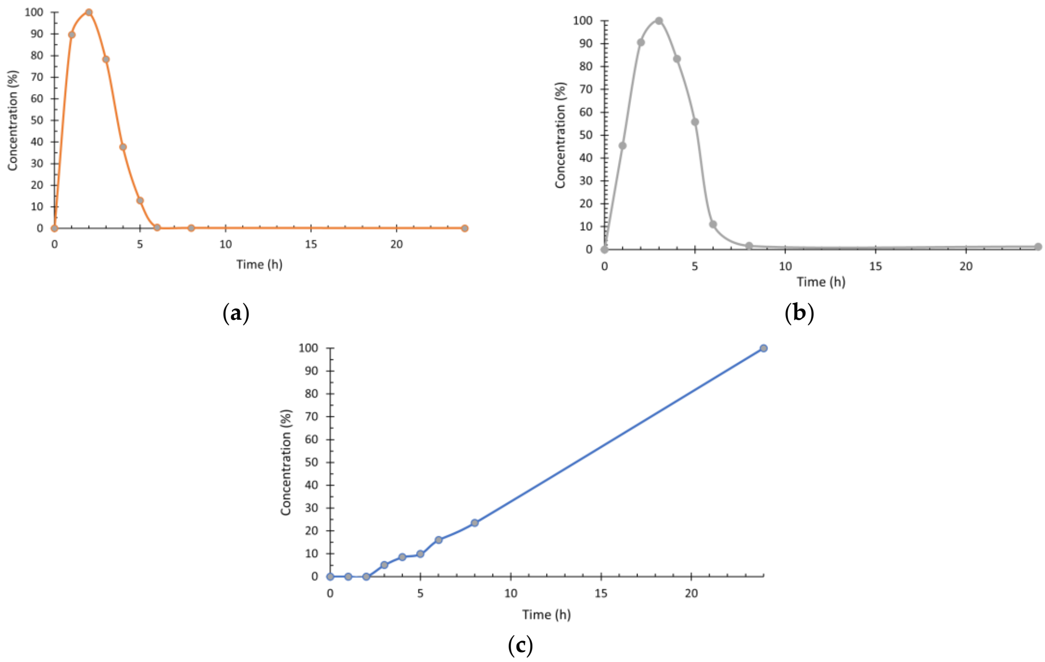

Figure S5. Concentration of all the intermediates in different times during the MP degradation: (a) intermediate A (b) intermediate B (c) intermediateC.

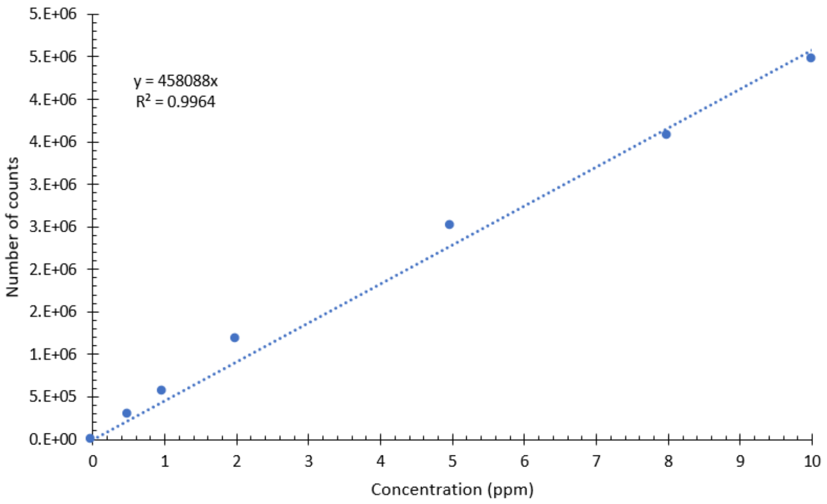

Figure S6. Standards of the 4-hydroxybenzoic acid (m/z = 136.8914).

Table S4. Concentration (ppm) of 4-hydroxybenzoic acid during the degradation of MP.

Time (h)	Area (counts)	Concentration (ppm)
0	0	0
1	0	0
2	0	0
3	60193	0.13
4	117756	0.26
5	101221	0.22
6	191869	0.42
8	279784	0.61
24	1193188	2.60
